# Applicability of a Novel Wearable Wireless Electrocardiogram Monitoring Device (Spyder) for Arrhythmia Detection in Patients with Suspected Cardiac Arrhythmias

**DOI:** 10.1155/2021/8496351

**Published:** 2021-11-26

**Authors:** Do Van Chien, Nguyen Thanh Binh, Nguyen Dung, Pham Truong Son

**Affiliations:** Department of Cardiology, Heart Institute, 108 Military Central Hospital, Hanoi, Vietnam

## Abstract

**Introduction:**

In clinical practice, many cardiovascular symptoms can be caused by arrhythmias that are not detected by electrocardiography (ECG) or 24–48 h Holter ECG monitoring.

**Aims:**

To describe the efficacy and applicability of a new device (Spyder) in detecting cardiac arrhythmias with midterm ECG monitoring.

**Methods:**

A descriptive, prospective study was performed on 26 consecutive patients who underwent midterm ECG monitoring with the novel ECG patch device (Spyder). The study was conducted over a 6-month period from August 2020 to February 2021.

**Results:**

Twenty-six patients (mean age, 57.8 ± 12.5 years; men, 77%) wearing a Spyder wireless ECG-monitoring device were recruited. The mean wearing time was 84 hours. The main indications for using the device were detection of recurrent atrial fibrillation after radiofrequency ablation (30.7%) and screening for atrial fibrillation after cryptogenic stroke (23.1%). All ECG monitor recordings obtained during the study period were of good quality. The device detected 12 episodes of atrial fibrillation in eight patients, one episode of ventricular tachycardia, one supraventricular tachycardia event, one case of paroxysmal third-degree atrioventricular block, and five cases of frequent premature ventricular contraction. The time to detection of the first episodes of atrial fibrillation and ventricular and supraventricular tachycardia was 28.8 and 47 hours, respectively.

**Conclusions:**

The new wearable wireless ECG-monitoring device (Spyder) is a feasible and effective method for the detection of cardiac arrhythmias.

## 1. Introduction

The worldwide spread of the coronavirus disease 2019 (COVID-19) pandemic and the resultant infections and deaths have necessitated changes in routine cardiology practice. Wireless devices and remote technologies may help cardiologists minimize direct contact between healthcare personnel and patients, which is an integral step in limiting the spread of COVID-19. Detection of arrhythmias poses a major challenge for cardiologists in this regard, and ambulatory electrocardiography (ECG) monitoring should be considered in patients with suspected cardiac arrhythmias [1]. In clinical practice, the 24 h Holter ECG is commonly used to monitor cardiac-related symptoms. However, it can only detect approximately 1%–5% of arrhythmias, and a number of patients required longer periods of hospitalization to identify the cause of the arrhythmia, which can increase the risk of COVID-19 contamination. Continuous ECG monitoring for more than 24 h is preferred to detect more silent heart rhythm disorders [2], and external ECG recorders have been shown to be noninvasive and more feasible with high efficacy. Of these, transtelephonic ECG event monitors (e.g., AliveCor and Apple watch) record only snapshot (symptomatic) events, usually over a duration of 30–45 s. In contrast, patch-based ambulatory ECG-monitoring devices record longer ECGs for up to several days or weeks. A number of devices (Zio Patch, SEEQ MCT, and Mypatch) have been shown to be useful for patch-based ambulatory ECG monitoring [3]. However, these devices are still not officially recommended for clinical practice because they often indirectly use pulse signals to record arrhythmias.

In Asia, some commercial devices have received approval, but they have been evaluated only in limited studies with small numbers of patients. Dr. P. Wong at the Singapore National Heart Centre invented Spyder, a wireless, patch-based ambulatory ECG-monitoring device. The Spyder is a lightweight, single-lead ECG device with no external leads or wires that allows for continuous ECG monitoring for up to 30 d. The recorded ECG data are wirelessly transmitted to a secure server without the user leaving their home. This device has been used in clinical practice at several cardiovascular centers; however, clinical experience with this novel device is currently limited, and it has not yet been approved by the FDA. Therefore, we conducted this study to evaluate the applicability of this device for detection of cardiac arrhythmias with midterm ECG monitoring.

## 2. Methods

### 2.1. Study Design

A prospective, cross-sectional study was performed using hospitalized patients and outpatients with suspected cardiac arrhythmias who were referred to the Department of Cardiology, Heart Institute, 108 Military Central Hospital, between August 2020 and February 2021.

### 2.2. Inclusion Criteria

We included patients aged over 18 years of age who (1) had the ability to use smartphones to communicate with investigators during the study period and comply with the study requirements, (2) had symptoms suggestive of cardiac arrhythmia or suspected atrial fibrillation within 7 days after the onset of cryptogenic cerebral stroke or 1 d after radiofrequency (RF) ablation, (3) wore the device for a minimum of 24 h and a maximum of 7 days, depending on the frequency of their symptoms, and (4) provided signed written informed consent.

### 2.3. Exclusion Criteria

Patients who had acute diseases requiring intervention or hospitalization, could not use smartphones or had unstable hemodynamics, or were allergic to ECG electrodes were excluded from this study.

### 2.4. Study Design

**Figure 1:**
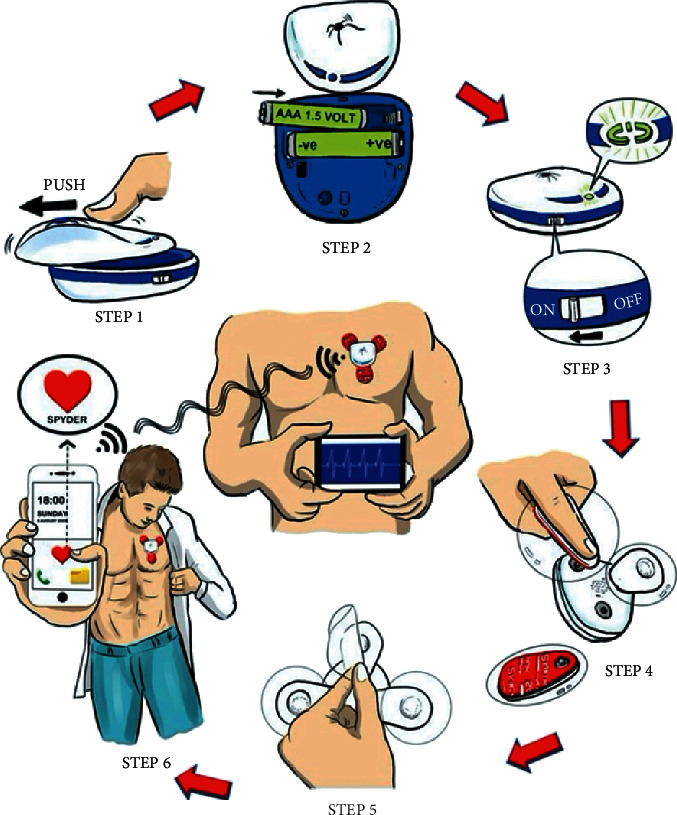
Instructions for usage of the Spyder ECG-monitoring device.

All patients were fully informed about the benefits and risks of participating in the study. A detailed clinical examination, blood pressure measurement, and electrocardiography were performed before the patients wore the device. Inpatients started wearing the device as soon as they achieved a stable status in the wards. The physician researcher trained the patients and/or their relatives until they could use the device comfortably. The instructions provided by the researcher are described in Figure 1 and can be summarized as follows: step 1: the device is opened to check it; step 2: the patient is instructed to replace the new 3 A battery for 3–5 days of use; step 3: the device is turned on to evaluate its operation; step 4: the adhesive electrode is inserted into the device and the patient is instructed on the method for changing the electrode; step 5: the glass stickers are peeled off; and step 6: the device is applied on the left chest and the connection with the phone is checked.

Spyder connects to the phone via Bluetooth, so the mobile phone should be kept close to the body to avoid losing the signal. The device receives signals from the heart and then transfers it to the mobile phone, which in turn transmits the ECG signal to a server over the Internet with the domain name https://www.doctorspyder.com. Thus, the patient's arrhythmias can be monitored in real time. Before hospital discharge, all patients were asked to practice using the device a few times until they were able to manage it independently (patients who had undergone RF ablation or stroke were trained during their hospital stay). Patients who showed severe symptoms were required to make a phone call to the doctors for advice and undergo hospitalization if necessary. Upon completion of the study, the device was sent back through a post office or a shipper.

### 2.5. Arrhythmia Criteria

Arrhythmia events were defined by the detection of any of the following findings: ventricular tachycardia, ventricular fibrillation, supraventricular tachycardia, sinus arrest (duration >3 s), supraventricular tachycardia (>4 beats, not including atrial fibrillation or flutter), atrial fibrillation (>30 ms), atrial flutter, atrioventricular (AV) block (second-degree 2 : 1, or third-degree AV block requiring advanced evaluation by the investigators), and ventricular tachycardia (VT) (>4 beats).

### 2.6. Statistical Analysis

The Statistical Package for Social Science software (version 22.0; IBM SPSS 22, SPSS Inc., Chicago, USA) was used to analyze the data. Continuous variables are presented as means and standard deviations, and categorical variables are presented as numbers and percentages. In comparisons of quantitative variables by Student's *t*-test, *p* < 0.05 was considered to be statistically significant.

## 3. Results

### 3.1. Clinical Characteristics of the Study Population

General characteristics of the study participants are presented in Table 1. A total of 26 patients were included in the study; the average age was 57.8 ± 12.5 years, and 77% of the patients were males (*n* = 20). Young patients (age, <40 years), patients aged 40–60 years, and those aged over 60 years accounted for 7.6%, 46.2%, and 46.2%, respectively, of the study population. The most common risk factors were hypertension (38.5%) and obesity (body mass index (BMI) > 25 kg/m^2^) (34.6%). The mean wear time was 84 h.

### 3.2. Clinical Indications for the Wearable Wireless ECG-Monitoring Device

Indications and wear time are presented in Table 2. Of the 26 patients, 8 (30.7%) were referred for detection of atrial fibrillation recurrence after RF ablation, 7 (27.7%) for dizziness or syncope, 6 (23.11%) for palpitations, and 7 (26.9%) for cryptogenic stroke or transient ischemic stroke. All ECG patch monitor recordings were of good quality, and no dermal irritability was recorded during the study period. The mean device wearing time for all indications was almost 84 h (range, 48–120 h).

### 3.3. Arrhythmias Detected by Spyder

The detected arrhythmias are presented in Table 3. Analysis of ECG-monitoring data showed 12 atrial fibrillation/atrial flutter episodes in eight patients, one episode of ventricular tachycardia in one patient, and one episode of supraventricular tachycardia in one patient. A third-degree A-V block was recorded in one patient. Frequent premature ventricular contraction (PVC) was recorded in five patients.

### 3.4. Time to the First Detection of Arrhythmias after Wearing the Spyder Device

Timing of arrhythmia detection is presented in Table 4. PVCs, which are common arrhythmias that occur in the first 24 h, were first detected within approximately 7.2 h of wearing the device. One case each of ventricular tachycardia and supraventricular tachycardia were identified after 47 h, and all cases of atrial fibrillation were detected after 28.8 h. No atrial fibrillation episodes were detected within 24 hours. All 12 atrial fibrillation/atrial flutter episodes were paroxysmal, with the duration of the episodes ranging from 30 s to 67 min. Among the 12 atrial fibrillation/atrial flutter episodes, only four were patient-triggered, while the remaining episodes were asymptomatic.

## 4. Discussion

In this study, we evaluated the applicability and effectiveness of the Spyder wireless ECG device in patients showing cardiac symptoms suspected to be caused by arrhythmia and those who had undergone post-RF ablation. All recordings with the Spyder device after correction were of good quality with no noise and were suitable for analysis. The time for which the device was worn ranged from 120 h and at least 48 h. The electrode adhesive pads used in the present study were consumables and needed to be replaced after 24 h of usage. The ECG patch in other ECG-monitoring devices (Zio Patch) can be used without changing the electrodes, which may have worn out because of the humid climate in Asian countries. During midterm Spyder usage with pad changes, no dermal irritation was found and good-quality ECG data were obtained, indicating that the changeable pad may be well tolerated and effective for continuous monitoring in Asian countries.

**Figure 2:**
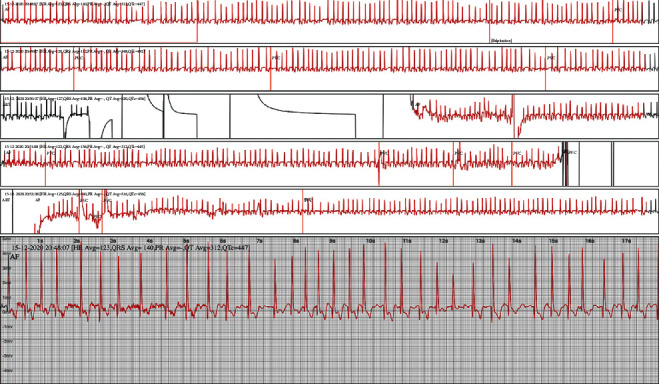
An episode of atrial fibrillation detected by Spyder in a patient with cryptogenic stroke after 28.8 hours of wearing.

The most common indication for ECG monitoring in this study was the detection of atrial fibrillation recurrence after RF ablation. The ability to identify atrial fibrillation is particularly important for patients who have undergone RF ablation, since atrial fibrillation recurrence is often paroxysmal and asymptomatic in such patients (Figure 2). The identification of recurrent atrial fibrillation can facilitate delivery of appropriate medical therapy, including anticoagulation, and provide important information regarding the rate and rhythm control in such patients. During the study period, 12 atrial fibrillation episodes (in all eight patients) were found, and these patients continued to receive medical treatment, including novel oral anticoagulants, as stated in the guidelines.

Approximately one-third of ischemic strokes are attributable to atrial fibrillation-mediated embolism [4], and the risk of recurrent thromboembolism is high if atrial fibrillation is undetected and untreated. Therefore, prolonged monitoring (up to 72 hours) to screen for atrial fibrillation in poststroke patients has been recommended in the guidelines. Initially, a 24 h Holter ECG was performed for all poststroke patients, and only one case with atrial fibrillation episodes was detected (Figure 2). In comparison with other studies [5], Spyder showed equivalent ability to detect atrial fibrillation disorders in patients after cryptogenic stroke. Detection of atrial fibrillation after stroke depends on the monitoring device used, the duration of the monitoring period, stroke type, and patient characteristics; thus, the rate of atrial fibrillation detection has been heterogeneous. A preliminary report on screening of atrial fibrillation in 363 patients in Singapore showed that atrial fibrillation could be detected in 4.1% of patients with a wearing time of 5.4 days [6]. Thus, the Spyder device was shown to be a good device for screening and detecting atrial fibrillation.

Whenever patients experienced symptoms, as trained, they pressed the button to mark the timing and notify doctors to monitor the software's server. Their ECG was then checked to confirm the diagnosis, and the decision to continue monitoring or hospitalize the patient was made depending on the severity of the arrhythmias. Previous studies have shown that doctors' clinical decision making in response to cardiac arrhythmias can be changed by using such remote electrocardiographic monitoring devices [7].

**Figure 3:**
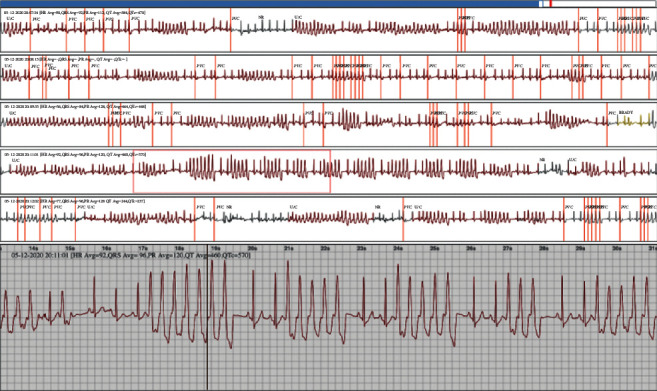
Nonsustained ventricular tachycardia detected by Spyder after 47 hours of wearing.

In our study, one patient showed ventricular tachycardia after 47 hours (Figure 3), and one showed supraventricular tachycardia after 47 hours that had not been detected by using a 24 h Holter ECG in the past. These two patients then underwent successful RF ablation. A paroxysmal third-degree AV block was detected 74.4 hours after wearing the device, and the patient underwent implantation of a permanent pacemaker the next day. Five cases showed symptomatic, frequent PVC; they were treated either with medical therapy (2 cases) or RF ablation (3 cases). Thus, the initial results from the Spyder device helped clinicians provide appropriate treatment for patients.

One of the advantages of this wireless device is that it is very compact, which reduced patient discomfort during motion. Moreover, the device could be removed when the patient showered without affecting the quality of ECG records. The researchers required only approximately 1-2 hours to guide the patient through proper usage of the device without a doctor's supervision. Moreover, in comparison with traditional 3–5-lead Holter ECG devices, the Spyder device allowed real-time detection of arrhythmias instead of a replay. However, the major limitations of the device were that patients were required to be familiar with smartphone usage, and they had to limit bathing while wearing the device. Moreover, assessment of arrhythmias required more time due to the long ECG recording time.

### 4.1. Study Limitations

This was a prospective, single-center study with a small number of patients. The study population was also not uniform, since many patients were unable to use smartphones, particularly elderly patients.

## 5. Conclusions

The new, wearable, wireless ECG-monitoring device (Spyder) offers a useful and effective method for the detection of arrhythmia disorders in patients with suspected symptoms due to cardiac arrhythmias.

## Figures and Tables

**Table 1:** General characteristics of the study participants.

Clinical features		Mean ± SD	Percentage (%)
Sex	Male	20	77
	Female	6	23
Age (years)	18–40	2	7.6
	40–60	12	46.2
	Over 60	12	46.2
Average age (years)		57.8 ± 12.5	
Weight (kg)		64.9 ± 15.2	
Height (cm)		166.8 ± 13.4	

*Risk factors*			
Hypertension		10	38.5
Dyslipidemia		8	30.1
Diabetes		3	11.5
Smoking		3	11.5
BMI ≥25 (kg/m^2^)		9	34.6
Mean CHA_2_DS_2_-VASc score (for atrial fibrillation patients)		1.2 ± 0.5	
Average wear time (h)		84	

**Table 2:** Indications and wear time.

Indications	Number of patients (*n* = 26)	Average (h)	Minimum (h)	Maximum (h)
Syncope	2 (7.6)	84	72	96
Dizziness	5 (20.1)	81.6	72	96
Palpitations	6 (23.1)	84	48	96
Postcryptogenic stroke	6 (23.1)	84	48	120
Transient ischemic attack	1 (3.8)	84	84	84
After ablation of atrial fibrillation	8 (30.7)	84	48	96

**Table 3:** Arrhythmias detected by the Spyder device.

Arrhythmias	Number of detected events	Number of monitored patients	Indications
Ventricular tachycardia	1	1	Syncope
Ventricular fibrillation	0	0	Dizziness
Sinus arrest >3 s	0	0	Syncope
Supraventricular tachycardia	1	1	Palpitations
Degree II A-V block	0	0	Dizziness
Degree III A-V block	1	1	Dizziness
Atrial fibrillation	12	8	Recurrence after RF ablation
Atrial flutter	0	0	Recurrence after RF ablation
Premature ventricular complex	5	5	Palpitations

**Table 4:** Timing of arrhythmia detection.

Indications	Detection in the first 24 h	Detection in the first 48 h	Time to first detection (h)
Tachycardia	No	Yes	47
Ventricular fibrillation	No	No	No
Sinus arrest >3 s	No	No	No
Supraventricular tachycardia	No	Yes	47
Degree II A-V block	No	No	No
Degree III A-V block	No	No	74.4
Atrial fibrillation	No	Yes	28.8
Atrial flutter	No	No	No
Premature ventricular contraction	Yes	Yes	7.2

## Data Availability

Data can be made available on request through email to the corresponding author.

## Abbreviations

AV: Atrioventricular
BMI: Body mass index
ECG: Electrocardiography
PVC: Premature ventricular contraction
RF: Radiofrequency.
